# Predictive properties of the A-TAC inventory when screening for childhood-onset neurodevelopmental problems in a population-based sample

**DOI:** 10.1186/1471-244X-13-233

**Published:** 2013-09-25

**Authors:** Tomas Larson, Sebastian Lundström, Thomas Nilsson, Eva Norén Selinus, Maria Råstam, Paul Lichtenstein, Clara Hellner Gumpert, Henrik Anckarsäter, Nóra Kerekes

**Affiliations:** 1 Department of Clinical Sciences, Lund University, Malmö (TL; HA), Lund (MR), Sweden; 2 Center for Ethics, Law and Mental Health (CELAM), University of Gothenburg, Gothenburg, Sweden; 3 Swedish Prison and Probation Service, Research & Development Unit, Gothenburg, Sweden; 4 Gillberg Neuropsychiatry Center (GNC), University of Gothenburg, Gothenburg, Sweden; 5 Department of Clinical Neuroscience, Karolinska Institutet, Stockholm, Sweden; 6 Department of Medical Epidemiology and Biostatistics, Karolinska Institutet, Stockholm, Sweden

**Keywords:** Autism, Tics, AD/HD, and other Co-morbidities inventory, A-TAC, Screening, Mental disorders diagnosed in childhood, Co-morbidity, Cohort studies, Predictive value of tests, Sensitivity and specificity

## Abstract

**Background:**

Identifying children with childhood-onset neurodevelopmental problems (NDPs, defined here as autism spectrum disorders [ASDs], attention-deficit/hyperactivity disorder [AD/HD], tic disorders [TDs], learning disorders [LDs] and development coordination disorder), using easily administered screening instruments, is a prerequisite for epidemiological research. Such instruments are also clinically useful to prioritize children for comprehensive assessments, to screen risk groups, and to follow controls.

Autism–Tics, ADHD, and other Co-morbidities inventory (A-TAC) was developed to meet these requirements; here the A-TAC’s prospective and psychometric properties are examined, when used in a population-based, epidemiological setting.

**Methods:**

Since 2004, parents of all Swedish twins have been asked to take part in an ongoing, nation-wide twin study (The Child and Adolescent Twin Study in Sweden). The study includes the A-TAC, carried out as a telephone interview with parents of twins aged 9 or 12. In the present study, screen-positive twins from three birth year cohorts (1993–1995) were invited to a comprehensive clinical follow-up (blinded for previous screening results) together with their co-twins and randomly selected, healthy controls at age 15 (Total N = 452).

**Results:**

Sensitivity and specificity of A-TAC scores for predicting later clinical diagnoses were good to excellent overall, with values of the area under the receiver operating characteristics curves ranging from 0.77 (AD/HD) to 0.91 (ASDs). Among children who were screen-positive for an ASD, 48% received a clinical diagnosis of ASDs. For AD/HD, the corresponding figure was also 48%, for LDs 16%, and for TDs 60%. Between 4% and 35% of screen-positive children did not receive any diagnosis at the clinical follow-up three years later. Among screen-negative controls, prevalence of ASDs, AD/HD, LDs, and TDs was 0%, 7%, 4%, and 2%, respectively.

**Conclusions:**

The A–TAC appeared to be a valid instrument to assess NDPs in this population-based, longitudinal study. It has good-to-excellent psychometric properties, with an excellent ability to distinguish NDPs (mainly ASDs) from non-NDPs at least three years after the screening evaluations, although specific diagnoses did not correspond closely to actual clinical diagnoses.

## Background

Since the 1990s, childhood-onset neurodevelopmental problems (NDPs) have been increasingly recognized in child and adolescent, as well as adult, psychiatry. In the *Diagnostic and Statistical Manual of Mental Disorders* (DSM) [1], these conditions are described in the section “Disorders usually first diagnosed in infancy, childhood, or adolescence.” In this paper, NDPs refer to autism spectrum disorders (ASDs, comprising autistic disorder, Asperger’s syndrome, and pervasive developmental disorder not otherwise specified, including atypical autism), attention-deficit/hyperactivity disorder (AD/HD), learning disorders (LDs), tic disorders (TDs), and developmental coordination disorder (DCD). The disorders referred to as neurodevelopmental are genetically predisposed, are associated with disorders affecting the brain and its development, and feature deficient cognitive abilities that develop during early childhood. AD/HD is one of the most prevalent disorders of childhood, present in at least 5% of all school-age children, while about 1% meet the criteria for an ASD, 1.5% for an LD, and 1% for a TD [2]. The prevalence of DCD ranges from 1.5% to 20% depending on how “caseness” is defined (the high prevalence figures reflect the number of children who fail a standardized test of motor coordination [3]). Associated disorders and comorbidities are common across all NDPs, and there is a considerable overlap with many adult psychiatric diagnoses [4,5]. The course of NDPs is not stagnant, and it has been suggested that NDPs are not discrete entities at all; indeed, “it would be inappropriate to diagnose one problem and not consider the implication of the other(s)” [2]. It has been proposed that children with problems severe enough to warrant clinical examination suffer from an “early symptomatic syndrome eliciting neuropsychiatric clinical examination” (ESSENCE), which may later in life correspond to criteria for a specific NDP, or to any mixture or sequelae of NDP diagnoses. Children with NDPs have been shown to be at risk of developing various functional impairments, mental health problems, and/or other difficulties, in severe cases requiring life-long intervention from medical and social services [2,6].

The literature was long dominated by studies on specific clinical groups, but more recent studies have often been population-based and longitudinal, challenging many established notions about NDPs. There is still a need for longitudinal follow-up studies of representative samples that take a comprehensive view of the spectrum of NDPs. The co-existence of disorders and the development of one problem into another raise important research questions, such as the possibility of shared etiologies and risk factors associated with heterogeneous phenotypes [7].

There are today many established rating scales and clinical instruments to assess NDPs, but there is still a need for an easily administered screening tool that covers the whole field of developmental problems, and that assesses not only caseness, but also sub-threshold traits and overlapping conditions. Such an instrument must not only differentiate those who present with symptoms from those who do not, but it must also be brief and easy to use, score, and tabulate. The A-TAC was developed to meet these requirements. Features that make the A-TAC unique is its systematic assessment of virtually all major overlapping and/or associated problem constellations in child and adolescent psychiatry, without letting the mutual exclusion criteria of the DSM obscure the true degree of overlap between problems. It is structured according to separate modules and taps into different problem areas without diagnostic hierarchies. It yields not only dimensional scores of symptoms, but also perceived suffering and dysfunction, for a broad range of neurodevelopmental and psychiatric disorders, including ASDs, AD/HD, LDs, TDs, and DCD. It is fully structured and validated for administration by lay assessors, as well as by professions. Unlike many other instruments, it is validated for use over the telephone. It was primarily developed for large-scale epidemiological studies, and three previous validation studies of the A-TAC have been performed, two by researchers involved in the present study [8,9] and one by an independent research group who examined the ASD domain only [10].

The A-TAC is an open access instrument for researchers and clinicians in the field, and the original Swedish version has thus far been translated into English, French, and Spanish. The English version is available at the BMC Psychiatry website [9]. The A-TAC has previously shown good test-retest measures and excellent inter-rater reliability and construct validity [8-10], and convergent validity with the Child Behavior Checklist [11]. Hansson and co-workers [8] reported excellent screening properties for ASDs and AD/HD in a clinical sample. These were replicated by Larson and co-workers [9], and cut-off scores were established for ASDs, AD/HD, DCD, LDs, TDs, and the modules “Perception” and “Planning & Organizing” specifically for screening purposes and for establishing proxies for clinical diagnoses. All previous validation studies were based on clinical groups, i.e., children referred for clinical neuropsychiatric assessments who were interviewed and subsequently compared to actual diagnostic outcomes and to controls. The present study adds a new perspective to the validation of the A-TAC by using the instrument to screen a population-based cohort of children who were subsequently followed up by experienced clinicians blind to all previous screening information using a combination of several diagnostic instruments to conduct a state-of-the-art clinical assessment. The clinical assessments were performed three years after the initial screening, and included screen-positive children, their twin siblings, and randomly selected controls. The overall aim of the present study was thus to validate the diagnostic predictive properties of the A-TAC in a population-based cohort.

## Methods

### Subjects at baseline (CATSS-9/12)

The Child and Adolescent Twin Study in Sweden, CATSS (for an overview, see [12]), is an ongoing study that aims to track all twins born in Sweden from 1992 on. A telephone interview is conducted with the parents of all participating twins around the children’s ninth birthday. The first three years of the CATSS interviews were conducted with parents of 12-year-old twins as well, in order to increase the number of birth cohorts included in the study. As of January 2010, 8610 parents had responded for 17 220 individual twins (8787 boys and 8433 girls). The overall response rate was 80%. All subjects are protected by the informed consent process and are given the opportunity to withdraw their consent and discontinue their participation at any time. The screening is conducted over the telephone by a market research centre, “Intervjubolaget,” using a computerized version of the A-TAC inventory. All interviews are performed by laypersons, who are given just a brief introduction to research methodology and child mental health problems. The interviewers enter the responses directly into an electronic database. The average interview time for A-TAC in the CATSS is about 30 minutes.

### A-TAC

The A-TAC consists of 262 items that address clinical issues: symptom criteria listed in the DSM for NDPs [1], key items for other psychiatric disorders according to the DSM [1], and additional items from published questionnaires and clinical practice. The items are divided into 20 different modules: Motor Control; Perception; Concentration & Attention; Impulsiveness & Activity; Learning, Planning & Organizing; Memory; Language; Social Interaction; Flexibility; Tics; Compulsions; Feeding; Separations; Opposition; Conduct; Anxiety; Mood; Concept of Reality; and Miscellaneous (i.e. items addressing clinically specific problem areas not covered by the other modules: sleep, food fads, severe overweight, bodily functions, and substance abuse). The two modules Concentration & Attention and Impulsiveness & Activity correspond to the definition of AD/HD. Similarly, the modules Language, Social Interaction, and Flexibility reflect the ASDs. Each module includes a number of symptom items coded on a dimensional scale: 0 indicates the item does not apply; 0.5 indicates the item applies or has applied in the past to some extent; and 1 indicates the item applies or has applied in full. All modules except Miscellaneous are organized according to a “gate structure,” in which all interviewees are asked questions that address module-specific core symptoms. There are 96 gate items in all, and only if any of these is at least partially endorsed will the interviewer continue with more detailed symptom questions.

### Subjects at follow-up (CATSS-15/DOGSS)

**Figure 1:**
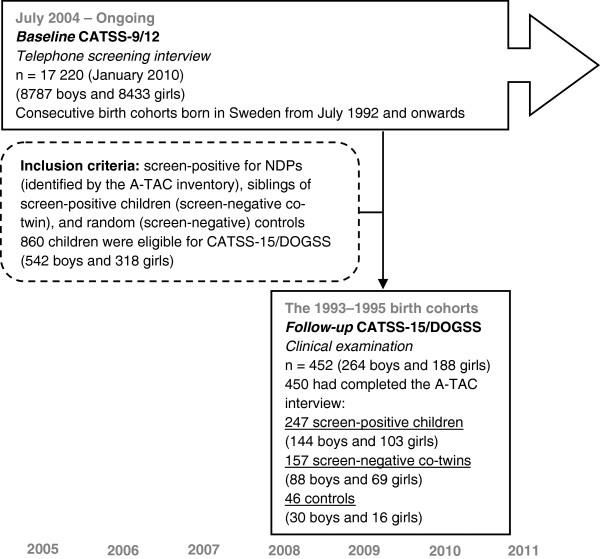
Flowchart for two of the phases in the CATSS study.

Twins born in the years 1993 to 1995 were eligible for the follow-up at age 15. Those born 1993 to June 1995 had been screened with the A-TAC at age 12, while those born in July to December 1995 had been screened at age 9. A sample of 15-year-olds was selected to create a population-based study group enriched for NDPs in the CATSS-15/DOGSS (Developmental Outcomes in a Genetic twin Study in Sweden) project to clinically assess the outcome of the A-TAC population screening for NDPs. Same-sex twin pairs in whom at least one of the siblings had screened positive for ASDs, AD/HD, TDs, LDs, DCD, and/or behavioural disorders with known NDP comorbidities, such as obsessive compulsive disorder (OCD), oppositional defiant disorder (ODD), conduct disorder (CD), and/or eating disorder (ED) were invited to participate (about 15% of the total population). In addition, a number of randomly selected population controls were included (5% of the total population). From the 1995 cohort, the inclusion criteria were narrowed to include ASDs and AD/HD only [12]. The final cohort selected by these criteria (referred to as the “follow-up” in this paper, see Figure 1) consisted of 860 individuals, of whom 452 (52%) consented to participate (247 screen-positive children, 157 screen-negative co-twins, and 46 randomly chosen controls matched for sex and age). For two of the included co-twins, full A-TAC information was lacking; these individuals were therefore not included in the longitudinal analyses.

### Procedures at follow-up

All screen-positive children and their siblings were diagnosed at age 15 according to the DSM criteria [1], using the Schedule for Affective Disorders and Schizophrenia for School-Age Children: Kiddie – SADS – Present and Lifetime Version (K-SADS-PL) [13] as the principal diagnostic interview. Each pair was assessed in one day by two specially trained psychologists who were blind to the previous screening results and performed their assessments independently, each seeing only one of the twins and a caregiver. In addition to the K-SADS-PL, the following diagnostic protocols and tests were used: the Asperger Syndrome (and high-functioning autism) Diagnostic Interview [14], the Wechsler Intelligence Scale for Children [15], the QbTest [16], and structured diagnostic assessment used by the Paris Autism Research International Sib pair study [17]. By interviewing the parent and the child individually, the interviewer was able to record separate interview scores for each diagnosis listed in the K-SADS-PL. Based on all sources of information available, the interviewer subsequently made a diagnostic summary rating according to the DSM criteria and the K-SADS-PL algorithms. As a final step, the clinician, together with a senior clinical expert (co-author ENS, a clinical board-licensed specialist in child and adolescent psychiatry), scrutinized the summary ratings for all cases assessed by the clinicians, with the aim of ascertaining definitive diagnoses based on all accessible information from the clinical evaluations. These consensus conferences were also conducted without access to the results of the screening interviews or the key to pairing the adolescents. For the ASDs, these diagnoses were further validated by a consensus on diagnostic criteria by several experts with access to all data except the scores from the baseline A-TAC interviews.

### Statistical analyses

The clinical diagnoses from the follow-up at 15 years of age (CATSS-15/DOGSS) were used as dependent variables, while the A-TAC scores from the CATSS-9/12 were used as independent predictors. Plotting sensitivity and specificity on a receiver operating characteristics (ROC) curve can help to determine the usefulness of the instrument and the optimal cut-off value (inflection point). On a ROC curve, the true-positive rate (sensitivity) is plotted against the false-positive rate (1 - specificity) for each possible threshold of the instrument. An ideal instrument will have a cut-off value that gives both a sensitivity of 1 (100% identification of true cases) and a specificity of 1 (100% exclusion of non-cases). Therefore, the score coming closest to this point determines the cut-off value. In reality, inventories have ROC curves between two extremes, and the greater the area under the curve (AUC), the more closely the instrument approximates the ideal. Hence, an AUC equal to 0.50 signals random prediction, an AUC of 0.60–0.70 indicates poor validity, 0.70–0.80 is fair, 0.80–0.90 is good, and AUC > 0.9 shows excellent validity [18]. The A-TAC cut-off values were determined and validated by two earlier studies [8,9] and used to assign screening status from the A-TAC scores.

First, the number of screen-positive children with a neurodevelopmental diagnosis identified in the screening phase was compared to the number of children who were given a diagnosis in the clinical examination three years later. Next, predictive psychometrics were calculated: first the AUCs and then the probabilistic measures of sensitivity, specificity, positive predictive value (PPV), negative predictive value (NPV), and diagnostic odds ratio (DOR), using both the “low” cut-off (optimal for screening purposes) and the “high” cut-off (allotted for use in research as a proxy for clinical diagnosis), both established in the earlier study [9]. PPV is the ability of the instrument to correctly identify children who truly have the condition, and NPV is its ability to correctly identify those without the condition. The DOR of a screening test is the ratio of the odds of those with the condition being screen-positive to the odds of the non-afflicted being screen-positive. The value of a DOR ranges from zero to infinity, with higher values indicating better discriminatory test performance. The DOR increases with the test’s ability to discriminate, and rises steeply when sensitivity or specificity becomes near perfect [19,20].

All statistics were calculated using the SPSS software package 19.0 and the online interactive statistical resource: StatPages [21].

### Ethical considerations

All participants consented to the study after receiving written and oral information. Analyses were performed on anonymized data files. The study protocol accorded with the Helsinki declaration and was approved by the ethical review board of Karolinska Institutet, Solna, Sweden (No. 02–289).

## Results

### A-TAC screening compared to diagnostic outcome

Among the 247 screen-positive cases, 198 children (80%) were screen-positive for an NDP: 27 children (14%) were screen-positive for ASDs, 95 (48%) for AD/HD, 74 (37%) for LDs, 35 (18%) for TDs, and 38 (19%) for DCD (See Table 1, Baseline. Note that the percentage total does not add up due to the overlap of diagnoses).

**Table 1:** Screen-positive cases according to A-TAC of screen-negative siblings and random, screen-negative controls compared with subsequent gold standard diagnoses at a clinical examination three years later

			**Follow-up Clinical Diagnoses in CATSS-15/DOGSS**						
**Baseline A-TAC in CATSS 9/12**			**NDP**^**A**^				**No NDP**^**A**^	**No NDP**^**A **^**Specified**	
			**ASDs**	**AD/HD**	**LD**	**TD**		**Other**	**No**
								**K-SADS**	**K-SADS**
								**diagnoses**^**C**^	**diagnoses**
Screen-positive cases according to the CATSS-15/DOGSS inclusion criteria N = 247			*N = 20*	*N = 72*	*N = 23*	*N = 47*	*N = 125*	*N = 42*	*N = 83*
	198 out of whom were NDP^A^ screen-positive	*of whom 27 were screen-positive for ASD*	*13*	*10*	*8*	*7*	*2*	*1*	*1*
		*of whom 95 were screen-positive for AD/HD*	*10*	*46*	*15*	*19*	*34*	*11*	*23*
		*of whom 74 were screen-positive for LD*	*5*	*23*	*12*	*8*	*38*	*12*	*26*
		*of whom 35 were screen-positive for TD*	*4*	*12*	*3*	*21*	*7*	*2*	*5*
		*of whom 38 were screen positive for DCD*	*3*	*3*	*6*	*4*	*26*	*5*	*21*
	49 out of whom were screen-positive for other mental health problems only^B^		*1*	*9*	*1*	*8*	*35*	*18*	*17*
Screen-negative siblings of cases N = 157			0	20	2	16	125	*32*	*93*
Screen-negative random controls N = 46			0	3	2	1	41	*13*	*28*
Missing data N = 2^D^			0	1	0	0	0	*0*	*0*
N = 452			N = 20	N = 96	N = 27	N = 64	N = 291	*N = 87*	*N = 204*

Baseline: CATSS-9/12 study (N = screen-positive in A-TAC).

Follow-up: Diagnostic outcome in the clinical follow-up study, CATSS-15/DOGSS (N = clinical diagnosis).

No NDP specified: Children who received another diagnoses in K-SADS or no diagnosis at the clinical examination (*italics* indicate those screen-positive, but do not add up due to diagnostic overlap).

^A^**NDP** defined as ASD and/or AD/HD and/or LD and/or TD and/or DCD (However, DCD had no corresponding diagnosis in the clinical examination), with a possible overlap of other mental health problems.

^B^**Other mental health problems** defined as OCD and/or ODD and/or CD and/or ED, with no NDP overlap.

^C^**Other K-SADS diagnoses** (i.e. “Other mental health problems” and/or depression, anxiety, stress disorder, mania, and/or psychosis with no NDP overlap).

^D^**Missing data**: one sibling was not screened at 12, but diagnosed with AD/HD at follow-up. Another sibling could not participate in either baseline or follow-up, due to somatic problems (Sotos syndrome).

Of the 27 children screen-positive for ASDs, 13 (48%) were diagnosed with an ASD at the follow-up, 12 (44%) were diagnosed with another NDP, 1 (4%) was diagnosed with a non-NDP mental health problem, and 1 (4%) had no DSM diagnosis at all (see Table 1, Clinical Diagnoses).

Of the 95 children who had screened positive for AD/HD, 46 (48%) were diagnosed with AD/HD at the follow-up, 15 (16%) with another NDP, 11 (12%) with a non-NDP mental disorder, and 23 (24%) had no DSM diagnosis.

Of the 74 who had screened positive for LDs, 12 (16%) were diagnosed with an LD at the follow-up, 24 (32%) with another NDP, 12 (16%) with another mental disorder (non-NDP), and 26 (35%) had no DSM diagnosis.

Of the 35 children who had screened positive for TDs, 21 (60%) were diagnosed with a TD at the follow-up, 7 (20%) with another NDP, 2 (6%) with another mental disorder (non-NDP), and 5 (14%) had no DSM diagnosis.

Of the 198 children who screened positive for any NDP at baseline, 108 (55%) received at least one clinical DSM diagnosis of an NDP at follow-up. Of the 252 children who were screen-negative for NDPs at baseline (the majority of whom were screen-negative co-twins to screen-positive siblings, and therefore constituted a high-risk group), 51 (20%) received at least one clinical diagnosis of an NDP, while 201 (80%) received no clinical NDP diagnosis at follow-up. A 2 × 2 contingency table of true and false positives and negatives for all NDPs yielded a sensitivity of 0.68, a specificity of 0.69, and a DOR of 4.7 for the A-TAC’s ability to capture any NDP (Table 1).

Among the screen-negative siblings, none were diagnosed with an ASD, 20 (13%) were diagnosed with AD/HD, two (1%) were diagnosed with an LD, and 16 (10%) were diagnosed with a TD.

Among controls, none was diagnosed with an ASD, but 3 (7%) were diagnosed with AD/HD, 2 (4%) with a TD, and 1 (2%) was diagnosed with an LD.

### Psychometric properties of the A-TAC in a three-year population-based follow-up

**Figure 2:**
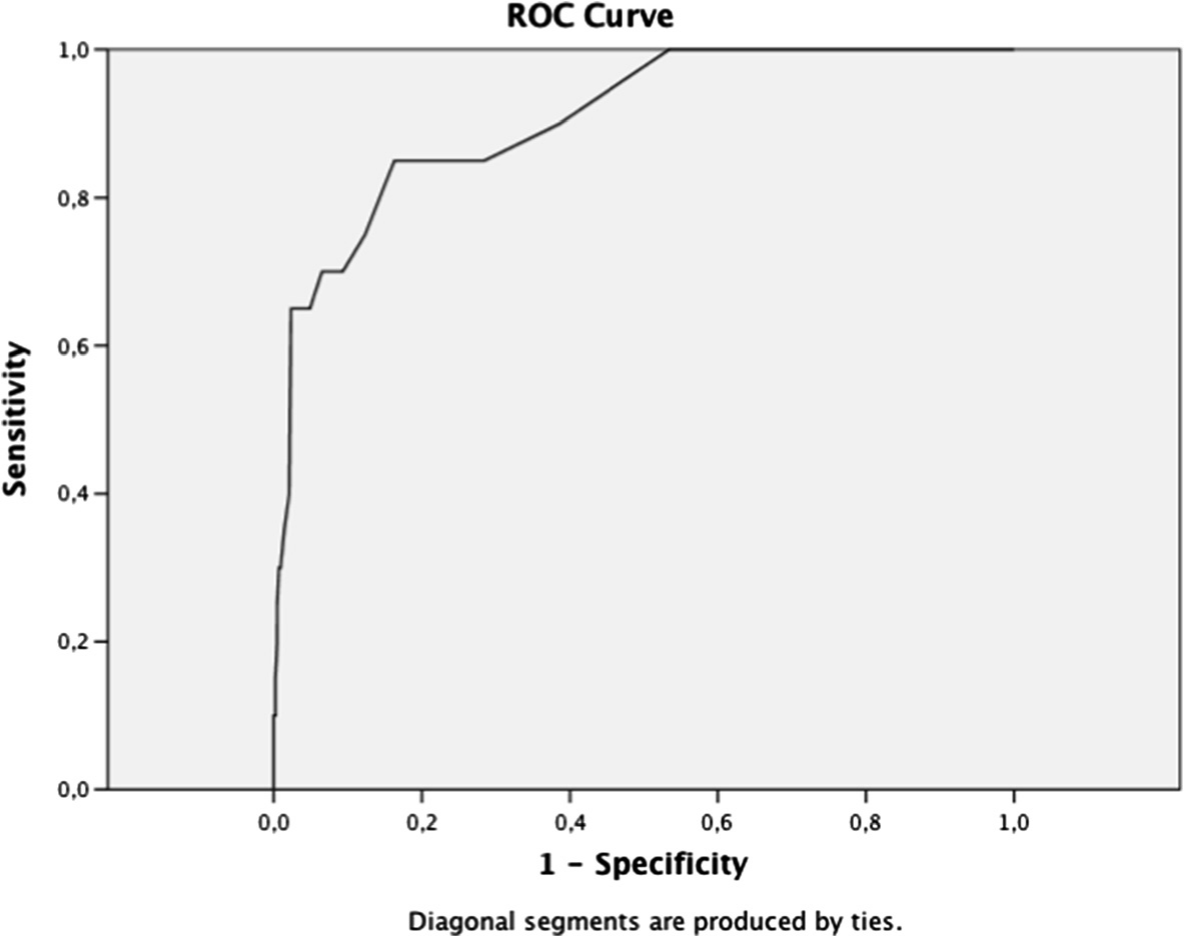
Receiver operating characteristics curve for autism spectrum disorders.

**Figure 3:**
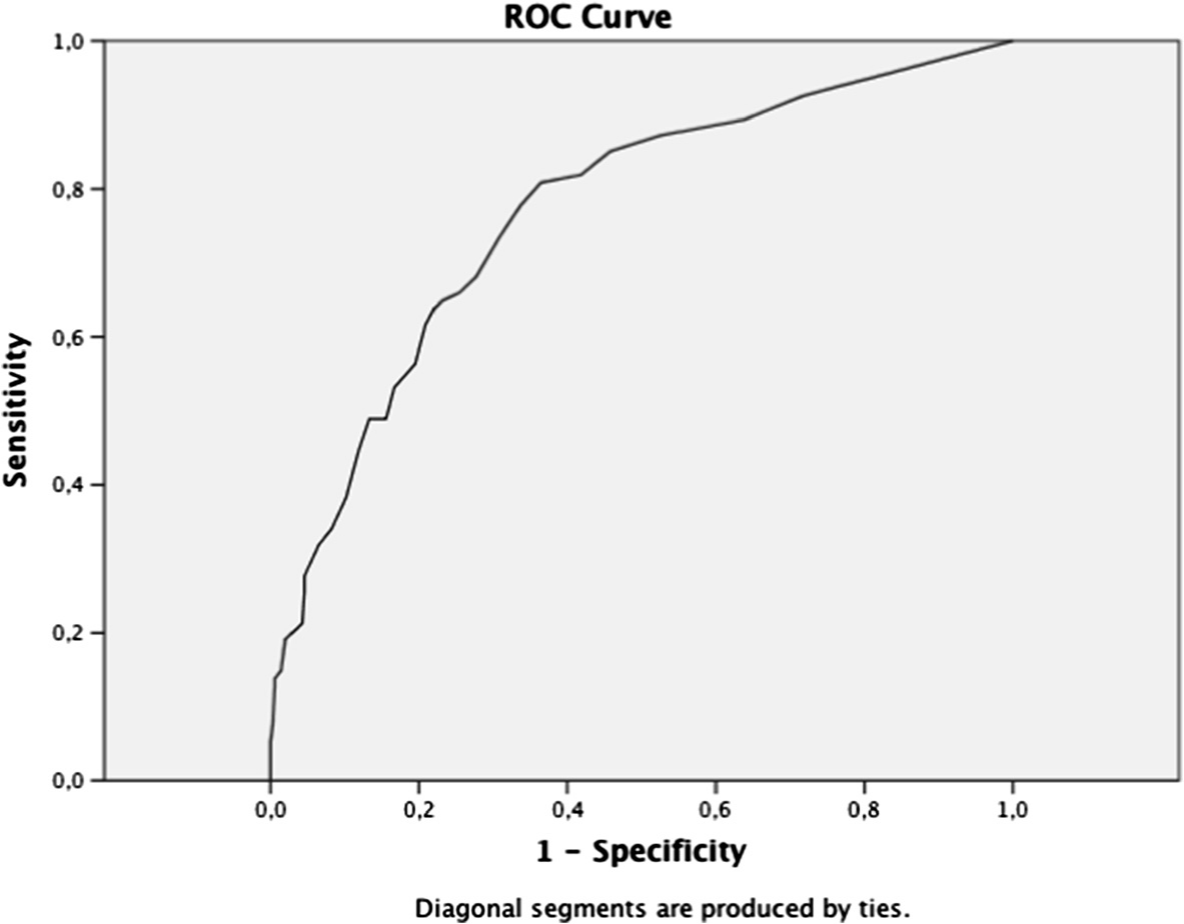
Receiver operating characteristics curve for attention-deficit/hyperactivity disorder.

**Figure 4:**
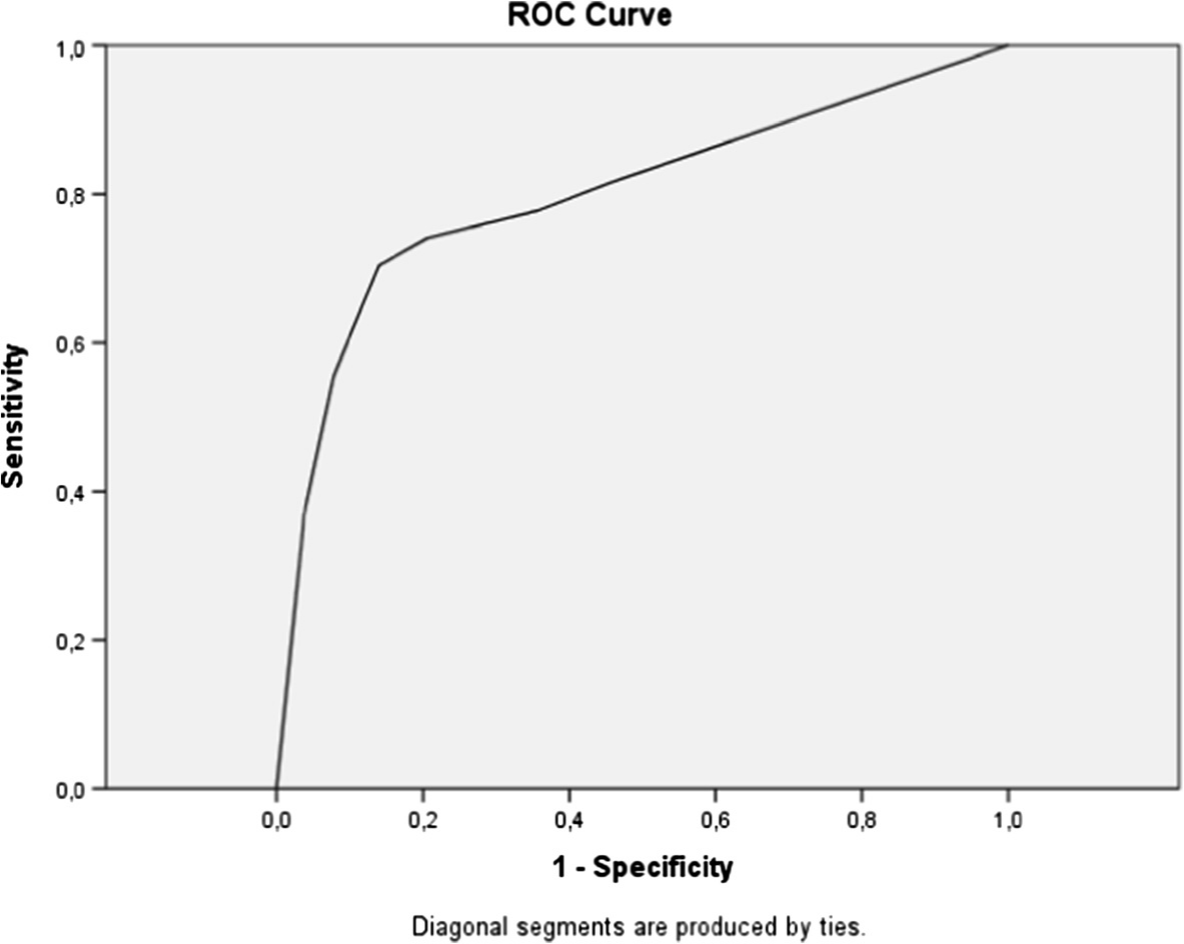
Receiver operating characteristics curve for learning disorders.

**Figure 5:**
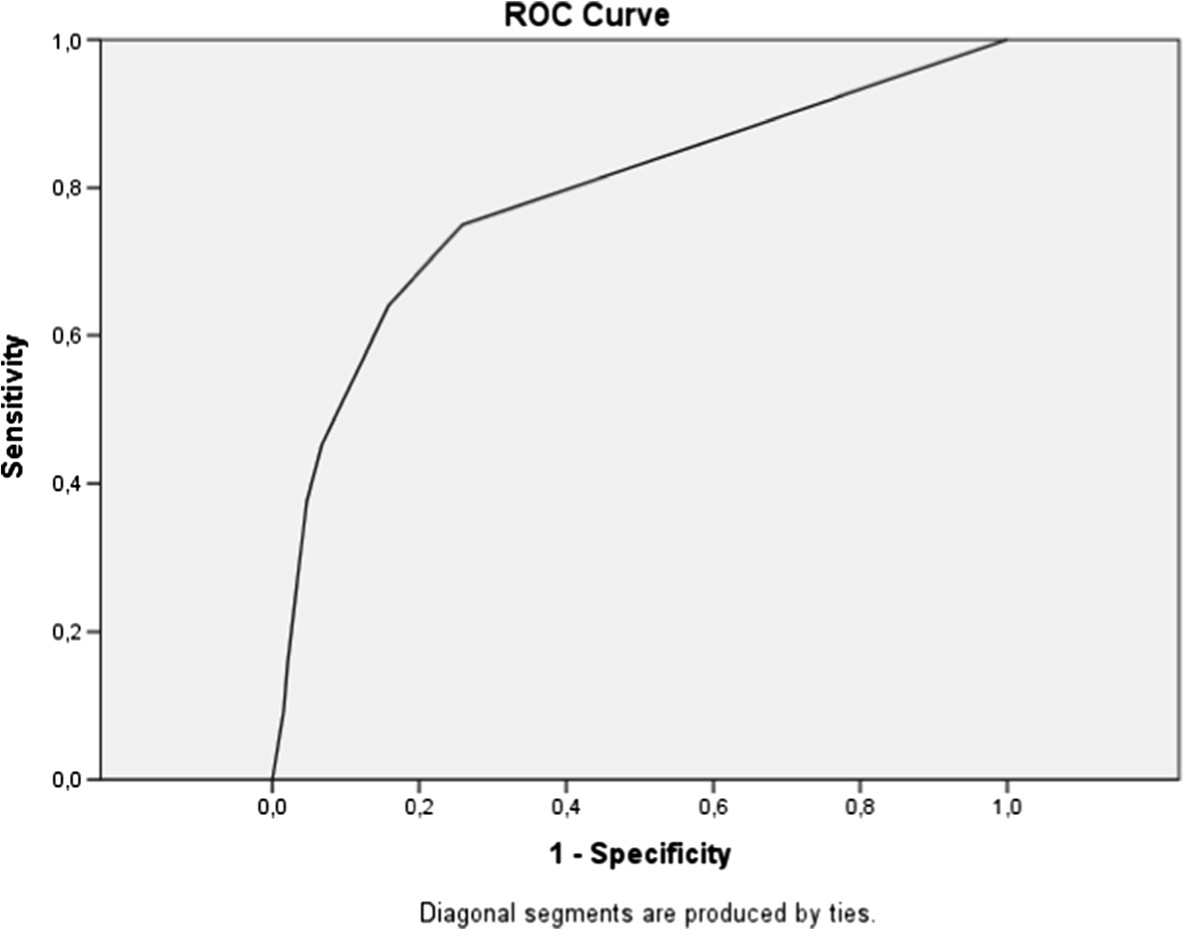
Receiver operating characteristics curve for tic disorders.

Predictive psychometrics are reported in Table 2, the AUCs are followed by the cut-off values, the low cut-off for screening possible caseness was used at baseline, and the supplementary high cut-off values for research diagnostic purposes are shown solely to complete the psychometric presentation. The ASDs domain in the A-TAC showed excellent predictive ability for clinical diagnoses of ASDs at age 15 (AUC = 0.91), followed by fair predictive ability for the AD/HD, LDs, and TDs modules (AUC = 0.77–0.80). Figures 2, 3, 4 and 5 illustrates the ROC plots for each of the targeted disorders. Overall, the low cut-off scores were coupled with higher sensitivity, while the high cut-off scores were, as expected, associated with better specificity. Specificity was generally higher than sensitivity, except for LDs. The low cut-off scores for each diagnosis showed a specificity and a sensitivity ≥ 64%. Table 2 shows the high cut-off scores, which had a specificity ≥ 93% for these disorders. The TDs module has just one cut-off value, with a sensitivity of 45% and a specificity of 93% in the general population group. ASDs yielded the highest DOR with a value of 29 using the low cut-off, and showed an even higher DOR (46) using the high cut-off. Among specific diagnoses, AD/HD and LDs showed the lowest DORs (6 for both) with the low cut-off, and more elevated values (8 and 15, respectively) using the high cut-off.

**Table 2:** Predictive psychometric properties for neurodevelopmental problems in the A-TAC inventory

	**AUC**	**CUT-OFF**	**SENS**	**SPEC**	**PPV**	**NPV**	**DOR**
**ASDs**	**0.91**	**4,5** (low)	**0.70**	**0.93**	**0.30**	**0.99**	**29**
		**8,5** (high)	**0.30**	**0.99**	**0.60**	**0.97**	**46**
**AD/HD**	**0.77**	**6** (low)	**0.64**	**0.78**	**0.44**	**0.89**	**6**
		**12,5** (high)	**0.20**	**0.97**	**0.63**	**0.82**	**8**
**LDs**	**0.80**	**1** (low)	**0.78**	**0.64**	**0.12**	**0.98**	**6**
		**3** (high)	**0.37**	**0.96**	**0.39**	**0.96**	**15**
**TDs**	**0.79**	**1**	**0.45**	**0.93**	**0.53**	**0.91**	**11**

Predictive psychometrics: area under the receiver operating characteristics curves (AUC), positive (PPV) and negative predictive value (NPV), and diagnostic odds ratio (DOR) for each diagnosis, depending on cut-off values (low/high) in the A-TAC.

## Discussion

About half of all A-TAC NDP screen-positive children had an actual clinical diagnosis of an NDP at the clinical examinations, and about 40% of those were the same NDP diagnosis that they had screened positive for three years earlier.

The coexistence of diagnoses (often referred to as “co-morbidity”) was more common than “pure” diagnoses. This finding fits with a growing body of evidence [2,7], suggesting that children with NDPs have very high rates of co-occurring problems. There is also an increasingly documented drift between the NDP categories. Several researchers have suggested that pure NDPs are relatively unusual [2,22,23] and that all children with an NDP should be assessed for all types of possible overlapping conditions.

The diagnostic outcome in this systematic clinical follow-up of a total population screened for NDPs showed that screen-negative children, even high-risk cases such as co-twins of affected children, generally did not have ASDs or LDs, but around 10% had sufficient AD/HD problems or TDs to meet the criteria for one of these diagnoses. It thus seems that the A-TAC is reasonably good at excluding NDPs in population studies, even though the total sensitivity for any NDP was just below 70%; a low score therefore is far from confirmation that no NDP-related mental health issues or diagnostic conditions are present.

In this prospective population-based study, the A-TAC once more had excellent screening properties for ASDs (AUC = 0.91, with a sensitivity of 70% and a specificity > 90%). These values are comparable to previously reported figures from clinical samples. However, the instrument was less accurate when screening for other diagnoses than ASDs (AD/HD, LDs, and TDs). Still, the low cut-off values showed a sensitivity around 70% (the TDs module represented the low point with 45%). Considering the interval of three years, these figures are still rather consistent, and conform to suggestions that about half of children diagnosed with AD/HD seem to grow out of it, at least in the sense that they no longer meet the diagnostic criteria for this particular condition [24], or that key AD/HD features may transform into other mental health problems [4,25].

In clinical practice, instruments with a high specificity result in under-referrals, but in epidemiological studies the aim is generally to screen out all the “normals.” This study aimed to establish the psychometric properties of A-TAC in a study group of children drawn from the general population. It was considered necessary to enrich the study group for NDPs in order to have sufficient power for these relatively rare conditions. The use of different cut-off points depends on the intended use of the scale. For two-stage investigations in which it is important that cases are recognized during the screening phase, because all positive cases will subsequently undergo clinical assessment, it is necessary to reduce the false negatives to a bare minimum. It is therefore preferable to use scales and cut-offs with a very high sensitivity, even though this usually compromises specificity. Nevertheless, the low cut-off values in A-TAC did not compromise specificity overall, except in the case of LDs (sensitivity 78% and specificity 64%). Thus, the earlier established low cut-offs worked well in this general population group to identify children who would be included with good cost-effect balance in clinical assessments.

In this population, negative predictive values were consistently high (≥ 89%), thus assuring the user that almost all children who screened negative did not meet diagnostic criteria for an NDP. A high rate of false positives is not uncommon in behavioural screening, which often yields low positive predictive values [26]. For this reason, the high cut-offs have been identified to serve as proxies for clinical diagnoses in epidemiological studies.

Using sensitivity and specificity alone as measures for an instrument’s efficiency can often be misleading. Sensitivity is only part of the discriminatory evidence, as high sensitivity may be accompanied by low specificity. Additionally, no simple aggregation rule exists to combine sensitivity and specificity into one measure of performance. For this, a single indicator of an instrument’s performance such as the DOR is required. The DOR is reasonably constant for a large range of cut-off scores on the ROC curve (see Table 2), but for the extremes of sensitivity and specificity the DOR rises steeply. If the original results in both NDPs and non-NDPs had followed a logistic distribution with equal standard deviation, the DOR would have been constant for all possible cut-off values. The DOR is thus a good measurement in meta-analyses of diagnostic studies that aim to combine results from different studies into summary estimates with increased precision [27].

Although the sensitivity and specificity of screening tools are affected by the prevalence of the disorders, they can also be influenced by differences in the characteristics of various disorders, such as clinical severity, and the characteristics of subjects, such as age and sex. For example, among girls, the lower rates of disruptive behaviour problems, along with a preponderance of inattentive symptoms relative to impulsive symptoms, may partially explain why NDPs often go unrecognized in girls [28].

Screening and diagnostic/identification tools that detect neurodevelopmental behaviour problems are good aids for clinicians, since they provide a structured and systematic assessment procedure that increases diagnostic reliability. There is, however, always a risk that a specific instrument is chosen because of its predominant standing in the field or in the literature, and not because it has the most accurate validation or otherwise is most suitable to the purpose.

All screening instruments should always be interpreted with caution. Omnibus assessment tools warrant critical attention, especially since they are important in research—not least in epidemiologic studies—because they provide prevalence figures in a population, make it possible to discern trends, and provide proxies for clinical diagnoses in scientific studies.

Clinicians and researchers often turn to a “broadband” assessment scale to ensure a comprehensive assessment of the presenting problem and to assist in the identification of co-morbid difficulties. There may be certain advantages in the extensive use of a particular scale, such as the ability to compare studies and the widespread familiarity with the scale among researchers and clinicians, but every scale has limitations that may remain unchallenged while worthy alternatives may be overlooked. Because most diagnostic measures for NDPs are designed specifically for categorical features, not broader phenotypes, there is also a need for instruments like the A-TAC that can provide more continuous measures of various aspects of NDPs.

The coexistence of diagnoses encountered in the field of NDPs points to the fact that developmental problem areas are not pure. In the general population, a dimensional/continuous distribution across diagnostic NDP categories has been reported [29].

The concept of ESSENCE was coined to account for this interrelatedness and coexistence of NDPs across diagnostic boundaries [2]. The disorders within the ESSENCE model are today diagnosed as separate categories, but they almost always overlap with each other, and can all be considered “neurodevelopmental” or “neuropsychiatric.” It is therefore vital that all early symptomatic syndromes eliciting neurodevelopmental clinical examinations are taken into account when looking for etiological/pathogenetic links, developmental trajectories, risk factors for negative outcomes, or interventions and treatments [30].

There is an essential need for broader NDPs screening instruments, but many screening tools are aimed mainly at strictly defined cases of childhood-onset disorders, and so are often likely to miss overlapping and associated disorders. The present study shows that the A-TAC instrument is useful as a broadband, first-level screening instrument in a population-based study group. Broadband screening tools for NDPs should generally be administered before narrowband screening instruments to ensure that common conditions, such as language impairment or learning disabilities, are detected.

### Limitations

Because study questions on diagnostic accuracy generally evaluate the association between inventory scores and health status, a cross-sectional design is a natural basic design option. However, this basic design has various modifications, each with specific pros and cons in terms of scientific requirements, burden for the study subjects, and efficient use of resources. In this case, a major factor that affects the instrument’s performance in relation to clinical diagnoses of NDPs is the time between the behaviour sampled and the clinical examination. There was a three-year delay between the parental assessment on the A-TAC and the clinical follow-up. Asking parents to rate current behaviour when symptoms of NDPs may be at their most prototypical, and then clinically examining the children three years later could have contributed to the difficulties in differentiating between NDPs (apart from ASD, which is one of the most constant NDPs), at least between the ages of 9 or 12 and 15. Even if some of the major child psychiatric problem constellations are established by age 12, the complex psychosocial problems associated with puberty that emerge around the time of the clinical examination may interfere with interpreting the results. Moreover, the A-TAC showed comparatively low DOR/AUC for disorders other than ASDs (especially AD/HD). This may be attributed principally to the time lag between the screening and the clinical assessment and perhaps also to a “twin sample bias” suggested to be inherent in using a screen-negative group that largely consists of genetically at-risk siblings. Given that NDPs are under complex and multivariate genetic influences and tend to follow a waxing and waning course, a longitudinal twin sample may compromise probabilistic measures, including NPVs and PPVs, since discordant co-twins will be more likely than other pairs to oscillate above or under a cut-off. Despite this reasoning, however, the notion of a twin sample bias is dubious since numerous studies have reported that twins differ only marginally from singletons [31-33]; even if same-sex twins may not be representative of the general population, is it unlikely that this circumstance would have had any substantial effects on the results presented here.

### Strengths of the study

The strengths of this study lie in its investigation of the efficacy of the A-TAC in a population-based cohort of screen-positive children, their screen-negative siblings, and controls and in its rigorous assessments of neuropsychological outcomes. Psychiatric interviews were carried out for all children in the study using the K-SADS-PL schedule, and consensus diagnoses were made by specially trained psychologists and an experienced child psychiatrist.

## Conclusions

The A-TAC is an effective method of first-level screening for NDPs, and it works well as a predictive assessment tool in the general population, particularly because of its ability to assess features and problem areas associated with ASDs.

## Abbreviations

AD/HD: Attention-deficit/hyperactivity disorder; ASD: Autism spectrum disorder; A-TAC: Autism–Tics, AD/HD, and other Co-morbidities inventory; AUC: Area under the curve; CD: Conduct disorder; DCD: Developmental coordination disorder; DOGSS: Developmental outcomes in a genetic twin study in Sweden; DOR: Diagnostic odds ratio; DSM: Diagnostic and Statistical Manual of Mental Disorders; ED: Eating disorder; ESSENCE: Early symptomatic syndrome eliciting neuropsychiatric clinical examination; K-SADS-PL: Schedule for Affective Disorders and Schizophrenia for School-Age Children: Kiddie – SADS – Present and Lifetime Version; LD: Learning disorder; NDP: Neurodevelopmental problem; NPV: Negative predictive value; OCD: Obsessive compulsive disorder compulsive disorder; ODD: Oppositional defiant disorder; PPV: Positive predictive value; ROC: Receiver operating characteristics; TD: Tic disorder

## Competing interests

The authors declare that they have no competing interests.

## Authors’ contributions

TL was involved in drafting the manuscript, collecting data, and conducting statistical analyses; SL in coordinating the study and conducting statistical analyses; TN, ENS, and MR in performing clinical assessments and revising the manuscript; PL, CHG, and HA in conceiving and designing the study and revising the manuscript; And NK in conducting statistical analyses, and drafting and revising the manuscript. All authors read, commented upon, and approved the final manuscript.

## Pre-publication history

The pre-publication history for this paper can be accessed here:

http://www.biomedcentral.com/1471-244X/13/233/prepub
